# Parameters for the Mathematical Modelling of *Clostridium difficile* Acquisition and Transmission: A Systematic Review

**DOI:** 10.1371/journal.pone.0084224

**Published:** 2013-12-20

**Authors:** Eroboghene H. Otete, Anand S. Ahankari, Helen Jones, Kirsty J. Bolton, Caroline W. Jordan, Tim C. Boswell, Mark H. Wilcox, Neil M. Ferguson, Charles R. Beck, Richard L. Puleston

**Affiliations:** 1 School of Community Health Sciences, University of Nottingham, Nottingham, United Kingdom; 2 Melbourne School of Population and Global Health , University of Melbourne, Melbourne, Australia; 3 School of Mathematical Sciences, University of Nottingham, Nottingham, United Kingdom; 4 NHS England Area Team Derbyshire, Nottingham and Nottinghamshire, United Kingdom; 5 Department of Clinical Microbiology, Nottingham University Hospitals NHS Trust, Nottingham, United Kingdom; 6 Department of Microbiology, University of Leeds, Leeds, United Kingdom; 7 School of Public Health, Imperial College London, London, United Kingdom; University of California, Davis, United States of America

## Abstract

**Introduction:**

Mathematical modelling of *Clostridium difficile* infection dynamics could contribute to the optimisation of strategies for its prevention and control. The objective of this systematic review was to summarise the available literature specifically identifying the quantitative parameters required for a compartmental mathematical model of *Clostridium difficile* transmission.

**Methods:**

Six electronic healthcare databases were searched and all screening, data extraction and study quality assessments were undertaken in duplicate. Results were synthesised using a narrative approach.

**Results:**

Fifty-four studies met the inclusion criteria. Reproduction numbers for hospital based epidemics were described in two studies with a range from 0.55 to 7. Two studies provided consistent data on incubation periods. For 62% of cases, symptoms occurred in less than 4 weeks (3-28 days) after infection. Evidence on contact patterns was identified in four studies but with limited data reported for populating a mathematical model. Two studies, including one without clinically apparent donor-recipient pairs, provided information on serial intervals for household or ward contacts, showing transmission intervals of <1 week in ward based contacts compared to up to 2 months for household contacts. Eight studies reported recovery rates of between 75% - 100% for patients who had been treated with either metronidazole or vancomycin. Forty-nine studies gave recurrence rates of between 3% and 49% but were limited by varying definitions of recurrence. No study was found which specifically reported force of infection or net reproduction numbers.

**Conclusions:**

There is currently scant literature overtly citing estimates of the parameters required to inform the quantitative modelling of *Clostridium difficile* transmission. Further high quality studies to investigate transmission parameters are required, including through review of published epidemiological studies where these quantitative estimates may not have been explicitly estimated, but that nonetheless contain the relevant data to allow their calculation. [Systematic review reference: CRD42012003081]

## Introduction

*Clostridium difficile* (*C. difficile*) infection (CDI) is a considerable public health problem with over 20,000 cases in the UK and up to 300,000 in the US annually [1]. In the last decade, new strains of and epidemiological changes in existing strains of the organism have emerged, in particular ribotype 027. This highly pathogenic ribotype has resulted in substantial morbidity and mortality [1-3]. CDI results in diarrhoea which ranges in severity from mild to severe, which in life threatening cases may require surgery [1] . Outbreaks of CDI have occurred in a wide range of healthcare settings including acute care hospitals, nursing homes, intensive care units, as well as in community settings. These have caused considerable political and public disquiet and have spurred government-driven action to address this organism both in the UK and internationally [3]. However, much remains unknown regarding the factors which influence CDI acquisition and transmission, therefore potentially compromising the development of effective interventions and control policies. 

Transmission of *C. difficile* from hospitalised, symptomatic cases was previously thought to be the primary source of disease; however a recent hospital based study has shown that transmission from these cases accounts for no more than 25% of new hospital cases [2]. Asymptomatic carriage or colonisation in both patients and healthcare workers, or infection from other community sources entering the hospital, may have relevance to propagation within the healthcare environment [4,5]. However, uncertainties in attributing acquisition to the community or from within the hospital setting, coupled with limitations in microbiological testing methods, complicates understanding of the routes of transmission and acquisition [6-8]. 

CDI has in recent years been noted among groups previously considered to be at low risk of acquiring the disease including young adults, pregnant women and people without apparent prior exposure to antibiotics or healthcare facilities [9]. The possibility of food-borne acquisition of *C. difficile*, through contact with companion animals, infants and aerosolised faecal material has been suggested [10-13]. 

It is apparent that the mechanisms of *C. difficile* transmission are complex. Mathematical modelling could be a useful tool to improve our understanding of CDI dynamics, as has been shown for other complex infectious diseases such as influenza [14]. Such models could make a valuable contribution to optimising CDI management and control; for example by providing theoretical frameworks to model and monitor the spread of infection, to improve the understanding of the underlying factors that trigger the development of epidemics from sporadic cases, to predict future trends and for testing the effects of intervention strategies. 

### Objectives

We undertook a systematic review to collate and summarise the available literature where the quantitative estimates of the mathematical parameters required to inform the development of a SEIRS (susceptible, exposed [pre-infectious], infectious, recovered [immune], susceptible [second susceptible]) compartmental transmission model for CDI are explicitly stated. 

## Methods

This review was carried out in accordance with PRISMA guidelines. A completed PRISMA checklist is available (Table S1). The full study protocol is registered with the National Institute for Health Research international prospective register of systematic reviews (PROSPERO) - registration number: CRD42012003081 [15]. Minor subsequent protocol amendments were submitted to clarify the study populations and eligibility criteria. This systematic review of the mathematical parameters needed to model CDI is a necessary prerequisite to the development of theoretical frameworks that can represent the infection dynamics of this organism. A further systematic review of the epidemiological characteristics (infection rates and risk factors) of CDI will also be required.

### Search strategy and study selection

We searched six electronic databases: EMBASE (1980-2012), Medline (1946-2012), PubMed (1920-2012), Web of Science (1899-2012), CINAHL (1968-2012) and the Cochrane database of systematic reviews to identify all epidemiological studies and evidence based reviews assessing transmission and acquisition of CDI. Further publications and grey literature were identified through internet searches of relevant websites (World Health Organisation, European Centre for Disease Prevention and Control, UK Health Protection Agency, UK Department of Health, US Centres for Disease Control and Prevention, Public Health Agency of Canada, Centre for Health Protection, Hong Kong, National Institute of Infectious Diseases Japan and Chinese Centre for Disease control and Prevention) [15]. Keywords relating to ‘*Clostridium difficile*’, ‘epidemiology’, ‘transmission’ and each of the relevant mathematical parameters were used to identify relevant papers. Search terms were developed through discussion and consensus and piloted in each individual database before the formal search process. Where available, medical subject headings (MeSH) were defined for the population and outcome parameters and adapted in accordance with the specifications of each search engine. 

The final electronic search was performed on 8 October 2012. In addition, we performed reference and citation tracking of all included papers to further identify unpublished literature. No year limitations were applied. Studies in languages other than English were only considered if an English abstract was available.

### Eligibility criteria

Studies meeting all the following key criteria were included in the review:

1. Study design: Any experimental and observational studies assessing *C.difficile* transmission or acquisition
2. Population: persons of any age with laboratory-confirmed CDI whether symptomatic or asymptomatic
3. Outcome measures: studies specifically reporting parameters required to create a SEIRS type mathematical model of CDI, i.e. basic reproduction number, net reproduction number, incubation period, and contact pattern, force of infection, serial interval and duration of infectiousness, recovery rate and recurrence rates [16]

After excluding duplicates, a three-stage filter process (assessment of titles, abstracts and full text) was used to screen all identified studies against the eligibility criteria. At each stage the screening was managed using Endnote X4 ® (Thomson Reuters, California, USA). Screening was undertaken independently by two out of three reviewers (AA, HJ and EHO) with consensus by discussion and provision for arbitration by a third reviewer. 

### Data extraction

Data were extracted independently by two reviewers from each included study using a pre-defined piloted template. Disagreements were resolved through discussion with provision for arbitration by a third reviewer. Data extracted on study characteristics comprised design, country, time period, objectives, number of participants and method of subject selection, diagnostic method and inclusion/exclusion criteria. Data obtained on population characteristics were; subgroup studied, study setting, age, sex, co-morbidities and race/ethnicity. Outcome measures extracted were; basic reproduction number, net reproduction number, incubation period, contact pattern, force of infection, serial interval and duration of infectiousness, recovery rate, recurrence rate and definition of recurrence. No further data were requested from authors of included studies. (Definitions of the mathematical terms are listed in Table S4).

### Quality assessment

The quality of included studies was was assessed using only the Newcastle Ottawa Scale (NOS) for observational studies since no experimental studies were found. Consensus was reached by discussion, with arbitration by a third reviewer as necessary [17]. Studies were judged on three domains i) selection criteria of subjects, ii) comparability of subjects (adjustment for confounders) and iii) ascertainment of the exposure or outcome of interest, with a maximum total score of 9 for cohort or case-control studies and 7 for cross-sectional studies. A high score across all three assessment domains (participant selection, adjustment for confounders and ascertainment of outcome) suggests the study is of higher quality than those scoring at lower points on the scale. The paucity of information available from abstract-only manuscripts precluded assessment of quality for these studies. 

### Summary measures

Both the quality and data extraction findings were tabulated and synthesised qualitatively using a narrative approach in accordance with the framework described by the Economic and Social Research Council and recommended by the University of York Centre for Reviews and Dissemination. 

## Results

### Study Selection

**Figure 1:**
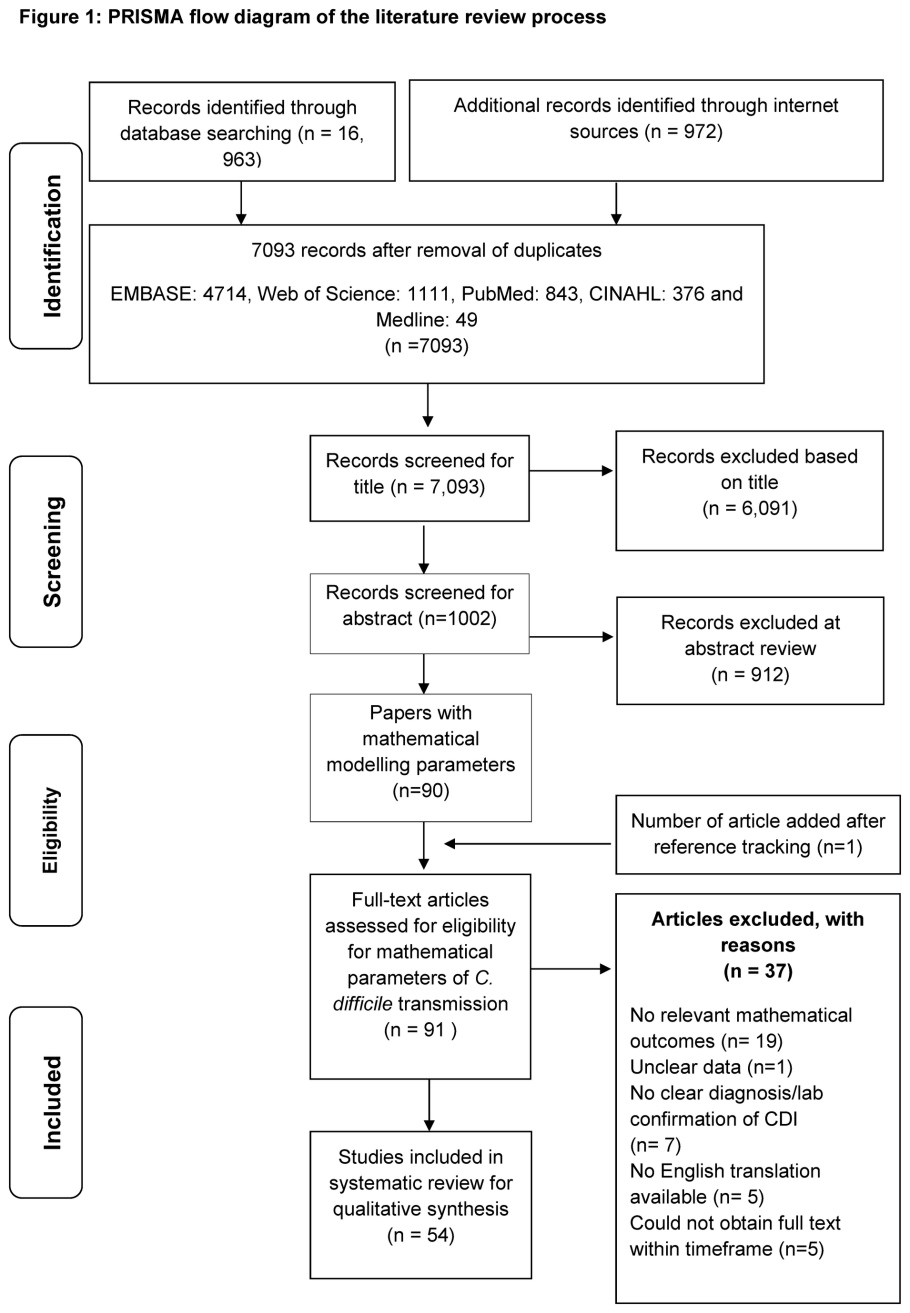
PRISMA flow diagram of the literature review process.

We identified 17, 935 articles; 16,963 from healthcare databases and the rest from other sources (Figure 1). After de-duplication, reference tracking and exclusion of studies that did not fit the inclusion criteria (at title and abstract review), 91 studies reporting mathematical modelling parameters for CDI were identified for full text review. A further 37 articles were excluded (Figure 1). Two of the included articles were from a single retrospective study but reported outcomes from different population subgroups so both were separately retained [18,19]. Overall, 54 full text articles were included in the narrative synthesis. 

### Description of included studies

The characteristics of the 54 studies which met the protocol eligibility criteria are summarised in Table 1 with further detail provided in Table S2. All identified studies were observational (55% [n=32] cross-sectional, 26% [n=14] cohort, 11% [n=6] case-control studies and in 4% [n=2] the study design was unclear). Data were reported on at least 26,137 subjects with CDI (the numbers included in one study were unclear). The number of subjects with CDI in individual studies ranged from 9 to 14,329 and four studies each made use of large hospital laboratory databases with over 1,000 CDI cases [20-23]. 

**Table 1:** Summary of study characteristics for included studies (N=54).

***Characteristic***	***Number of studies***	***% of studies***
**Study design**		
Cross-sectional studies	32	59
Case-control studies	6	11
Prospective cohort studies	14	26
Design unclear	2	4
**Country (UN inequality-adjusted Human Development Index 2010)**		
Very high	47	87
High	1	2
Medium/low	0	0
Data unclear	6	11
Study population/specific subgroups *		
Hospital inpatients (general/unspecified)	34	63
Hospital outpatients (general/unspecified)	20	37
Nursing/long-term care facility	5	9
All community cases within a circumscribed area	3	6
Renal patients	2	4
Organ/stem cell transplant patients	6	11
Other – HIV, neurology and psychiatric gerontology, surgery / gastroenterology, appendectomy, burns, gynaecological oncology, IBD	8	15
**Method of laboratory diagnosis ^*@*^**		
Toxin detection / immune assay	51	94
Stool culture	18	33
Polymerase chain reaction (PCR) ribotyping	6	11
Antigen detection	1	2
Multilocus sequence typing	1	2
Laboratory-diagnosed, methods unclear	2	2

^*^ Numbers do not sum to 54 as some studies include more than one population group

^@^ Numbers do not sum to 54 as some studies include more than one method of diagnosis

The characteristics of populations investigated varied substantially. Studies were reported from at least 17 different countries including USA (n= 23), Europe (n=13), South Korea (n=6) and Canada (n=3). There was wide age variability across most study populations (age range: 1 month to 99 years) but some focused solely on children or adults [19,22,24-31]. The majority of studies (n=34) included hospital in-patients with no other specified co-morbidities, but 16 were from within specific specialties or investigating patients with defined medical conditions. Whilst 21 studies investigated non-specific populations of hospitalised patients alone, the rest either combined both inpatients and outpatients, hospital inpatients with nursing home residents or patients diagnosed with CDI within a circumscribed geographical area [18-21,23,32-37]. 

### Quality assessment

The majority of studies included were judged to have low scores on the NOS. Among cross-sectional studies, scores ranged from one to seven [37,38]. In the case-control and cohort studies, scores ranged from three to seven [25,33,35,39-41]. Inadequate matching and adjustment for confounders was responsible for the lower quality assessment scores attained. Only two cross-sectional studies and one cohort study reported having adjusted for other confounding variables [38,42,43]. For most case-control studies, use of hospital control subjects resulted in lower quality assessment scores because NOS defines that only the use of community controls warrants a positive score since hospital controls may have other co-morbidities that could influence study outcomes. Although 4 out of 6 case control studies attempted to match hospital controls to cases by sex, month of diagnosis, antibiotic exposure or propensity score, it was unclear whether this was adequate to control for bias [32,44-46].

Other methodological concerns which resulted in poor quality assessment scoring were related to insufficient information on follow-up – 10 of 14 cohort studies, inadequate definitions of study outcomes - particularly for studies reporting recurrence and use of suboptimal testing techniques for CDI detection.

For 5 studies, no quality assessment could be performed because a full English translation of the article was unavailable [47-51]. For one study in which the study design was unclear, the quality assessment was made assuming it was a cross-sectional study [52].

### Synthesis of Results

#### Reproduction number

Only one hospital-based study reported estimates for the reproduction number based on a non-linear transmission model [42]. Another used ribotyping to estimate strain-dependent numbers of secondary cases per index case (Table 2) [34]. The study by Lanzas et al comprised 11,406 patients across 6 medical wards and was used to estimate parameters of a compartmental mathematical model for *C. difficile* transmission [42]. Hosts were classified as susceptible if they had received antimicrobial treatment. Those who had not been on antibiotic treatment were presumed to be resistant to colonisation. Infectious hosts were either diseased (*D*) (i.e. manifesting CDI), asymptomatically colonised with protection (*C+*) (mounted an immune response) or asymptomatically colonised without protection (*C-*) (no immune response mounted) [42]. The basic reproduction number (R_0_) (defined in this context as the average (median) number of secondary colonisations (*C+ or C*-) per colonisation (*C+ or C*-) or infection (*D*) in a ward free from CDI) was estimated at 1.04 (range: 0.55-1.99), with each host (susceptible *C+* or *C*- or infected *D*) generating on average 0.4 colonised hosts without protection (*C-*) and 0.6 colonised hosts with protection (*C+*). 

**Table 2:** Studies providing limits on basic reproduction number.

**Author**	**Year**	**Study details**	**Study Period**	**Basic Reproduction Number Data**
Lanzas [42]	2011	A mathematical model for *C.difficile* transmission which identifies hosts who have received antimicrobial treatment as susceptible, and distinguishes between diseased, asymptomatically colonised hosts with protection and asymptomatically colonised hosts without protection, was fitted to a hospital data set comprising 11046 patients in which diagnosis was by stool toxin	January to December 2008	Estimated basic reproduction numbers for variation in other parameters: Mean= 1.07, Median= 1.04. Range 0.55-1.99. New colonisations produced by asymptomatic or symptomatic patients’ averaged 0.4 new patients colonised without a protective response and 0.6 new patients colonised with a protective response
Noren [34]	2004	330 isolates from patients with toxin-positive diarrhoea were analysed by PCR ribotyping Secondary cases were linked to index cases using PCR ribotyping	February 1999 to January 2000	For ribotype SE17 (UK ribotype 012), 7 index cases gave rise to 19 secondary cases. Mean: 2.6 secondary cases per index case (range 1-7). For ribotypes other than SE17: Mean 1.2 secondary cases per index case (range 1-4)

Noren et al reported higher rates of secondary cases than Lanzas (335 *C. difficile*-infected patients from three hospitals [two tertiary care, one primary care]), although without accounting for non-linear transmission effects or the proportion of patients asymptomatically colonised, these estimates cannot be directly mapped to R_0_ [34,42]. The reported secondary case rates were strain-specific. Amongst the 8 strains identified, Swedish ribotype SE 17 (UK ribotype 012) appeared to pose the highest risk of transmission (mean 2.6 secondary cases per index case [range 1 to 7]). *C. difficile* types 11 (081) and 12 (002/159/183) had the lowest secondary case rate (1 secondary case per index case), while ribotypes 7b (054), 20 (001), 21 (014/077/020/220), 21b (014/077/020/220) and 23a (258) generated at least 1.2 cases [34]. Both studies investigating the reproduction number of CDI scored highly on the quality assessments. (Swedish ribotype, UK ribotype taxonomy matching – Personal communication, T Akerlund, Swedish Institute for Communicable Disease Control, October 2013)

#### Incubation period

Two hospital-based studies with estimates of incubation periods for CDI were found (Table 3) [2,53]. Although the studies were substantially different in terms of study size, study period and diagnostic technique, each estimated similar incubation periods of <4 weeks for the most probable transmission links of all identified “index case - secondary case” pairs. In the study by Samore, all 12 in-patients studied over a 2 month period became symptomatic 3 - 28 days (median 19 days) after exposure to infection from a symptomatic index case [53]. Walker et al, reported estimated incubation periods for three types of possible transmission links, the most plausible directional potential transmission links: Median incubation period 18, (range 8–42) days, the most plausible links: 24 (10–61) days and all potential links: 33 (13–74) days [2]. Both of these investigations achieved high NOS quality assessment scores, although neither explored the effects of potential confounding factors; in particular it is possible that the incubation period could be influenced by other factors within the host. Both studies were undertaken in a hospital setting so limiting their generalizability to community associated infection.

**Table 3:** Studies reporting data on incubation period.

**Author**	**Year**	**Study details**	**Study Period**	**Incubation Period Data**
Samore [53]	1996	52 ‘index’ cases with diarrhoea and positive stool toxin assay were included; stool samples from hospital roommates, occupants of adjacent wards and the patient subsequently occupying the room were analysed for evidence of transmission	June to December 1992	Interval between onset of exposure and onset of symptoms in 12 symptomatic contacts: 3-28 days, median 19 days. Interval between onset of exposure and positive culture in 19 asymptomatic contacts: 1-20 days, median 5 days
Walker [2]	2012	Data of 218 enzyme immune assay positive patients from a hospital laboratory database were used to analyse potential ward-based contacts between patients	September 2007 to March 2010	Incubation periods calculated: 61% of patients <=4wks. 13% of patients >12wks. Medians 18-33 days depending on possible links. IQRs 8-74 days, depending on possible links

#### Contact patterns

The nature of the contact implicated in *C. difficile* transmission was reported in three studies; hospital ward-based contacts and contacts between household members [20,38,53]. Results are shown in Table 4. These demonstrated the likelihood that *C. difficile* could spread from an infected individual to their ward-based or household contacts. Information reported in the household study was limited to relative risks [20]. Pepin et al, showed that child contacts of an infected individual had a higher risk of being infected than spouse contacts (relative risk, child: 90.61 [95% CI: 33.89 - 487.64] vs. spouse: 7.61 [95% CI: 5.77-9.78]), however there were few child contacts on which this estimate was based [20]. Information on ward based contact was limited to the duration of contact that could facilitate transmission (adjusted hazard ratio per daily roommate exposure: 1.11 [95% C.I 1.03-1.19]) [38]. 

**Table 4:** Studies reporting data on contact patterns.

**Author**	**Year**	**Study details**	**Study Period**	**Contact Pattern Data**
Hamel [38]	2010	Multiple databases of 37697 patients used to identify 202 cases of CDI (identified by enzyme immunoassay for toxin A or B) and room-mate exposures.	April 2001 to March 2006.	Total roommate exposure was associated with CDI - Hazard Ratio (HR) total roommates/day 1.06 (1.00-1.12). After adjusting for confounders, HR=1.11 (1.03-1.19).
Pepin [20]	2012	2222 patients diagnosed with CDI were identified from a hospital database. Cases related to one another were found and verified using telephone number.	January 1998 to December 2009.	5/1061 spouses and 3/501 children developed CDI. Attack rates were 4.71/1000 and 5.99/1000 respectively.
Samore [53]	1996	52 ‘index’ cases with diarrhoea and positive stool toxin assay were included; stool samples from hospital roommates, occupants of adjacent wards and the patient subsequently occupying the room were analysed for evidence of transmission.	June to December 1992.	99 contacts were analysed; 12 had *C. difficile* diarrhoea, 19 were asymptomatically colonised. Typing showed that only 6 of these contacts (5 symptomatic, 1 asymptomatic) had the identical strain to the index case. 42% of the index cases had at least 1 positive contact.

 The study by Pepin et al (2012) achieved the lowest NOS quality score [20]. The authors were unable to prove donor-recipient linkages, and thus the strength of evidence for the reported risk for household contacts is questionable since ‘secondary infection’ in the household may not necessarily be attributable to the index household case.

#### Force of infection

No studies describing the force of infection were identified. 

#### Serial Interval

The serial interval of CDI was reported for household and hospital contacts in two studies (Table 5) [2,20]. There was some variability in reported intervals which may reflect differences in study settings and methods. One study suggested that the serial interval of CDI in a hospital setting is likely to be <1 week but in some circumstances could be up to 8 weeks [2]. The second study reported serial intervals in household settings ranging from 6 to 50 days and in one situation up to 186 days [20]. Although the lower limits reported in the second study correspond to that of the first, this study utilised a small cluster of cases and achieved a low NOS quality score. 

**Table 5:** Studies reporting time interval between infection and onward transmission.

**Author**	**Year**	**Study details**	**Study Period**	**Serial Interval Data**
Pepin [20]	2012	2222 patients diagnosed with CDI were identified from a hospital database. Cases related to one another were found and verified using telephone number.	January 1998 to December 2009.	9 household contacts with CDI: 8/9 secondary cases developed within 2 months of the index case.
Walker [2]	2012	Data of 218 EIA positive patients from a hospital laboratory database were used to analyse potential ward-based contacts between patients.	September 2007 to March 2010.	Minimum infectious period: 65% of transmissions ≤ 1wk, 82% ≤ 4wks, 10% > 8wks. Medians 1-8 days depending on possible links. IQRs 0-33 days, depending on possible links.

#### Recovery rate

The recovery rate from CDI was reported in eight studies (Table 6) [22,26,31,32,51,54-56]. Recovery was typically dependent on treatment with antimicrobials (either metronidazole or vancomycin). Two studies reported data on immune-compromised patients and both found similar recovery rates of 98% and 100% [31,56]. Five studies reported data on non-specific groups of hospital inpatients with recovery rates ranging from 75% to 94%. The lowest recovery rate of 56% was found in the only study which estimated recovery for both patients who had received any treatment for CDI and those who received none. Other studies typically estimated separate recovery rates for treated and untreated patients. Three of the studies were judged to have low NOS quality scores due to one or all of the following reasons: unclear definitions of recovery, lack of information on how recovery was ascertained and insufficient follow up data [31,54,55]. Of the others, three were judged to be of good quality on the basis of clear definitions and the use of consistent methods of ascertaining recovery [22,26,32].

**Table 6:** Studies reporting data on recovery rate.

**Author / Year**	**Year**	**Study subgroup**	**Study Period**	**Recovery Rate Data**
Alanazi [31]	2012	Pediatric patients undergoing hemopoietic stem cell transplantation	2001-2009	Responded to therapy with metronidazole: 78/80 (98%).
Bilgrami [56]	1999	Blood stem cell transplant patients	Mar 1993- Aug 1996	Recovered after initial therapy with metronidazole and/or vancomycin: 14/14 (100%) - 1 patient had a relapse but recovered afterwards.
Bouza [51]	1995	Hospital inpatients	1994 (one year)	Recovered after initial therapy (oral vancomycin or metronidazole): 76/83 (92%)
Khan [26]	2012	Hospital inpatients (≥15 years)	2006-2009	Cure rate (resolution of diarrhoea by day 6 of treatment, and negative assays on days 6 and 10): 96/123 (78%).
Kim [55]	2011	Hospital inpatients	Sep 2008-Jan 2010	Diarrhoea stopped without treatment: 49/189 (26%). Clinical cure rate (with treatment): 118/140 (84%). ‘Global cure’ i.e. cured without recurrence: 93/140 (66%).
Kim [22]	2012	Adult hospital inpatients (>18 years)	2004-2008	2008 cohort - ‘Improved' without therapy other than discontinuing antibiotics: 235/1367 (17%) – (NB the 1367 quoted includes patients who went on to have treatment). ‘Improved' with oral metronidazole: 796/846 (94%).
Kyne [54]	1998	Hospital inpatients	Jan-Jun 1995	Resolution of symptoms: 38/73 (52%). Of the 73 patients, 62 had had metronidazole or vancomycin therapy, 11 had no treatment.
Vesteinsdottir [32]	2012	All patients in Iceland (hospital-acquired and community-acquired CDI)	July 2010-June 2011	Recovery rate from primary CDI with 1 course of antibiotics: 70/93 (75%) – 69 patients had metronidazole, 1 had vancomycin.

#### Recurrence rate

Forty-nine studies reported recurrence of CDI data (Table S3) [18,19,21-30,32-37,39-41,43-52,54-70]. Rates ranged from 3 to 49% with variation by study population. Two studies investigated recurrence in circumscribed populations that comprised all hospital and community cases [32,34]. For studies considering only hospitalised patients, recurrence rates ranged from 3 to 36% were observed although it should be noted that the study reporting 3% was based on only 14 patients [59]. The highest proportion of recurrence was found among the elderly (35 - 39%), those resident in long-term care (up to 49%) or patients diagnosed with gastrointestinal related illnesses (45%) [48,58,60,69]. The generalizability of these findings is compromised by the variable definitions used for recurrence across the different studies.

#### Summary measures

No statistical pooling of results was undertaken because of the extensive heterogeneity of identified studies.

## Discussion

To our knowledge, this is the first systematic review undertaken to assess the evidence base on the mathematical parameter estimates required for modelling the infection dynamics of *C. difficile*. The study was designed to only search for studies that specifically estimated and reported on one or more of the quantitative parameters required to inform a SEIRs compartmental transmission model. Our summary of the evidence has identified that there is a paucity of literature available explicitly reporting the mathematical parameters which describe the transmission and acquisition of *C. difficile*. This probably reflects a lack of attention of the infectious diseases modelling community to this important disease. It is however likely that published epidemiological studies will contain suitable data from which these quantitative parameters can be estimated, even where these have not be explicitly stated. For example, the generation time and serial interval are composite quantities determined by the incubation period distribution and infectiousness over time in infected individuals. Nonetheless, the paucity of information available on the parameters required to construct suitable infectious disease models is an important finding in itself. Given the substantial burden posed by CDI, the near absence of published estimates with which to model CDI and therefore by implication the likely paucity of modelling activity is perhaps surprising and a missed opportunity. 

Limited evidence was identified on the reproduction numbers. A single study used a non-linear transmission model to estimate R_0_ and whilst point estimates for this threshold parameter were above one, the data were consistent with values below one [42]. However, it is important to note in this study by Lanzas et al, that the definition of colonization used by the research only includes those recently exposed to antibiotics. It therefore ignores those who may be carrying the organism asymptomatically but who nonetheless could be shedding it. Rates of secondary infection also indicated that transmission from infected patients alone may not sustain new cases of CDI within hospitals. This complements the results of a recent study by Walker et al (2012) that suggest that only about 25% of new hospital cases arise from patient-to-patient transmission within hospital wards [2]. Noren et al, reported that some strains appeared to be more likely to generate new cases than others [34]. Given experience with other infectious diseases, this finding is not surprising, e.g. meningococcal disease [71]. It is therefore possible that the transmission dynamics of *C. difficile* will vary by setting and strain. This has implications for the design of future studies undertaken to examine the transmission dynamics of *C. difficile*, as the findings may be strain and setting specific which could limit the generalizability and use of the data generated.

The incubation period of CDI was estimated to last between 3 and 28 days but in some instances up to 12 weeks. It should be noted that several sources of uncertainty exist when measuring the incubation period. Estimates typically depend on knowledge of the time / date of exposure which cannot always be ascertained, particularly for an infection which may be carried asymptomatically. Secondly, estimates are subject to the assumption that transmission arose from the first traceable contact. Even where typing studies show the same strain between patient contacts, there is still uncertainty as to whether transmission occurred as a result of direct patient contact or indirect contact with other potential sources. 

 The only conclusion that could be drawn on contact patterns is that with every additional day of ward contact with *C. difficile*-infected patients, there is an 11% increase in the risk of infection acquisition. There was no information on the frequency and intensity of contacts between patients or between members of the community that provided the opportunity for *C. difficile* transmission to occur. The information found on patient contacts would be insufficient to inform the contact WAIFW (who acquired infection from whom) matrix required for modelling, but is relevant to control policies which call for the use of isolation as an intervention to curtail the risk of *C. difficile* transmission in hospitals.

Serial intervals reported varied from less than one week to 186 days. Despite the variation in study settings, the lower limits of the serial intervals reported by both studies are comparable. This might be due to the fact that the risk of transmitting *C. difficile* between spouses in a house and patients in a shared ward depend on the same principles in terms of contact with contaminated surfaces. It should be noted that Pepin’s household study did not utilise molecular typing to confirm the identity of strains responsible for index cases and secondary cases so linkage cannot be assumed. This might explain the unusually long serial interval of up to 186 days estimated in this study [20]. 

There was a paucity of data on recovery. Observational studies do not always follow up all infected cases to record long term outcomes of infection, including complete recovery. Most of the studies included in this review relied on retrospective hospital data where recovery as an outcome may not have been documented. Had we aimed to look at the effects of the treatments used for CDI, more information on recovery rate may have been found, but that was beyond the scope of this review. 

Only two studies had adequate definitions of recurrence of CDI. These allowed for recurrence being due to either relapse of the primary infection or re-infection / new infection with a different strain [34,59]. The proportion of recurrences caused by these different possibilities will vary from cohort to cohort, potentially influenced by, for example, treatment efficacy and infection control precautions. Other (n=17) studies used the terms recurrence and relapse interchangeably and others did not clearly define these terms (n=27) [72]. 

The studies evaluated in this review were subject to methodological weaknesses which suggest that this field of research has some inherent difficulties. CDI is influenced by multiple potentially confounding factors (e.g. patient age, presence of co-morbidities, medication history and duration of inpatient stay), but these were not considered in many studies. Such residual confounding may partially explain some differences in findings among studies reporting similar parameters. All but thirteen studies estimated parameters using retrospective data [25,29,32,35,39,41,43,53,55,61-63,66]. This might explain inadequacies in outcome definitions in these studies since the data used were initially assembled for other purposes. Varying diagnostic techniques were used across all studies which further limits the generalizability of individual study findings. Diagnosis of CDI by looking for faecal *C. difficile* toxins only, has sub-optimal sensitivity and is generally considered to be insufficient as a stand-alone test for CDI. A combined two-test algorithm involving glutamate dehydrogenase preliminary screen or PCR for toxin gene, followed by toxin detection is recommended for use in England for greater sensitivity, specificity [73]. Standardisation of diagnostic approaches would be appropriate for future studies. 

### Strengths and limitations of this review

Our literature search was extensive with over 7,000 studies screened to identify potentially relevant articles. Study selection, data extraction and study quality assessment were all performed in duplicate to ensure consistency and reduce the potential for bias, particularly where subjective judgement was required. The authors conducted this review in accordance with PRISMA and the Centre for Reviews and Dissemination (CRD) guidelines. However, our ability to draw conclusions was limited by the number and quality of studies found, the limited reported detail and the heterogeneity of the included studies (which precluded statistical synthesis of the evidence to generate pooled estimates of parameters). The narrative analyses were limited mainly to epidemiological studies, and thus some of the findings (e.g. recovery rate) may not be comparable to results obtained from clinical trials on the effectiveness of various CDI treatments where definitions may differ. The differential use of transmission / mathematical modelling terminologies may have also limited our ability to identify all studies reporting the above parameters, although the extensive reference and citation tracking of relevant papers will have made this unlikely. This review was designed to identify only studies which specifically reported one or more of the quantitative estimates required. It is likely that this tight constraint will have excluded studies that report data which could be used to calculate these parameters but which don’t explicitly describe them. Recognising this, the next step of this work will be to systematically review the epidemiological study data in order to calculate these estimates. Nonetheless, this and further reviews may potentially be biased by the variability in how CDI is defined and diagnosed. The diagnostic methodologies have evolved over time with inconsistent sensitivity, specificity and predictive values which may compromise the comparability of results. This has implications for the development of mathematical models for CDI. For example, the incubation period assumes a date of inoculation resulting in infection; however this date is not necessarily clear for some infections, including CDI. It is thought that approximately 3% of the adult population carry the organism, but it is possible that these estimates are compromised by our current capacity to detect colonisation. Some of the included studies utilised data from children with apparent CDI as young as a month old. The validity of this could be questioned as colonisation without disease has been noted in as many as 70% of neonates [74]. However, pseudomembranous colitis associated with *Clostridium difficile* has been noted before in young babies [75].

A potential criticism could be that the research did not differentiate between mathematical parameters for community and hospital associated or acquired disease as the setting may influence the estimate for each parameter due to potentially different infection dynamics by setting. However, the purpose of the work was to elucidate all published estimates of infection parameters for *Clostridium difficile*, such that they could be utilised in models at a later date, which may or may not be adjusted to take account of setting according to the evidence on the importance of setting as a variable. It should be noted that recent research has suggested that our previous assumptions about the importance of ‘in hospital’ transmission and acquisition in the hospital setting have been thrown into doubt, given that only 25% of cases occurring in the hospital studied appeared to have an links to other cases in the hospital [2].

Our research was limited to human studies. It was beyond the scope of the work to consider animal based studies; however, this may have compromised the capacity to find suitable mathematical parameters as animal based models of the disease have been developed. Repeating this systematic review with animal models may be an area for future research.

## Conclusions

Mathematical models are increasingly being used to improve infectious disease control. The studies identified for this review suggest that the dynamics of human-human transmission of *C. difficile* are uncertain and provide insufficient evidence for creating a simple SEIRS type mathematical model of CDI. 

Well-designed prospective transmission studies are warranted. To establish transmission and acquisition parameters, including the serial interval, basic reproduction number and force of infection, studies would need to explore the linkage between primary and secondary cases. Given that this review has found that the reproduction rate may be different between strains, and that these can each be carried at variable rates in community and hospital settings, modelling studies may need to consider the possibility that strains can exhibit different transmission dynamics depending on the microbial burden and toxin concentrations they invoke in the host and any cross-strain protection that may be present. Additionally such studies may need to consider the setting. In seeking to elucidate the evidence base for the mathematical parameters that can be used to describe and model CDI, we have not differentiated between settings (e.g. acute hospital, community, care home). As this review has indicated, the transmission patterns of *Clostridium difficile* are not completely clear. Intuitively it might be assumed that they are different depending on the setting, however until the scientific understanding of the spacial and temporal relationships of organism acquisition prior to causing symptomatic infection are more clearly understood, this cannot be assumed. Nonetheless it may be appropriate to consider adjusting for setting in any future model development.

Future studies investigating CDI will necessitate clear, consistent definitions and molecular typing / whole genome sequencing. This is particularly relevant in the ascertainment of relapse versus re-infection and may also help to elucidate the prevalence, longevity and specificity of any immunity to *C. difficile*. Characterising population susceptibility and its modification with antimicrobial treatment, gut flora and IgG antibody to *C. difficile* strains is also important for interpreting reproduction rates in the modelling of outbreaks and the potential for this to vary between cohorts. CDI testing algorithms with higher sensitivity and specificity, positive and negative predictive value would improve the accuracy of diagnosis and hence the identification of *C. difficile*-infected individuals to inform these parameters. Future studies could include the screening of patients at hospital admission to determine rates of carriage and the propensity for hosts with asymptomatic carriage to infect others (however the positive and negative predictive value of testing methods becomes critical where the underlying prevalence is expected to be low). 

The need for more rigorous evidence on *C. difficile* transmission dynamics is particularly important given the burden of CDI. Further empirical evidence to quantify mechanistic transmission models could assist in controlling this organism and inform robust infection control or healthcare policy. Research in this area by experienced infectious disease modellers is overdue.

## Supporting Information

Table S1 **PRISMA checklist.**
(DOCX)

Table S2 **Characteristics of included studies.**
(DOCX)

Table S3 **Studies reporting data on recurrence rate.**
(DOCX)

Table S4 **Parameter definitions.**
(DOCX)
