# Supplementary material for: Parameters for the Mathematical Modelling of Clostridium difficile Acquisition and Transmission: A Systematic Review
Source: PLoS One. 2013 Dec 20;8(12):e84224. doi: 10.1371/journal.pone.0084224 (PMC3869946; doi:10.1371/journal.pone.0084224)
Supplement: Table S2 — Characteristics of included studies. (DOCX) [file pone.0084224.s002.docx]

**Table S2: Characteristics of included studies (n= 54)**

| Publication  Year | Country of study | Study design | Enrolment period (duration/dates) | Groups/sub groups studied | Age of subjects | Method of diagnosis | PCR / MLST used for epidemiological purposes | Number analysed in study (with *C. difficile*) | Outcomes reported |
| --- | --- | --- | --- | --- | --- | --- | --- | --- | --- |
| Alanazi, 2012 [[31](#_ENREF_31)] | Canada | Cross-sectional retrospective | 2001-2009 | Paediatric patients undergoing haemopoietic stem cell transplantation | Median 8 years (range 1-19) | Faecal cytotoxin | No | 83 | Recurrence of CDI |
| Alangaden, 2000 [[70](#_ENREF_70)] | USA | Cross-sectional  retrospective | Jan 1998-Jul 1999 | Haemopoietic stem cell transplantation | Not stated | Stool assay for cytotoxin A/B | No | 13 | Recurrence of CDI |
| Armbruster, 2012 [[37](#_ENREF_37)] | USA | Cross-sectional  retrospective | 2005-2009 | Inpatients and outpatients | Mean 58 years | Endoscopy /positive ‘C.difficile test’ from laboratory database | No | 210 | Recurrence of CDI |
| Aronsson, 1987 [[52](#_ENREF_52)] | Sweden | Unclear (observational) | 2 years (unspecified) | Patients with acute or chronic renal failure | Mean age 63 years (range 28-82) | Stool culture + stool cytotoxin assay (toxin B) | No | 62 | Recurrence of CDI |
| Barany, 1992 [[69](#_ENREF_69)] | Sweden | Cohort  unclear | 1984-1986 | Chronic or acute renal failure | Mean age 64 years (range 24-82years) | Stool culture and/or cytotoxinassay | No | 70 | Recurrence of CDI |
| Beausejour, 2010 [[43](#_ENREF_43)] | Not given | Cohort  prospective | 9 months (unspecified) | Presumed all hospital inpatients but not clear | Not stated | EIA of GDH confirmed by cell cytotoxicity assay | No | 86 | Recurrence of CDI |
| Berling,2009  [[68](#_ENREF_68)] | USA | Case-control  retrospective | Mar2005 - Nov 2006 | Nursing homes | Mean age cases: 75.9, controls 78.7 years. | Cytotoxin test | No | 40 | Recurrence of CDI |
| Bilgrami, 1999 [[56](#_ENREF_56)] | USA | Cross-sectional  retrospective | Mar 1993- Aug 1996 | Blood stem cell transplant patients | Mean age cases 45 (range 4 – 60) | Stool culture | No | 14 | Recovery Rate, Recurrence of CDI |
| Bouza, 1995 [[51](#_ENREF_51)] | Spain | Cross-sectional  retrospective | 1994 (one year) | Hospital inpatients | Mean 56.9 years (range 20 – 92) | Cytotoxin assay/culture |  | 120 | Recovery Rate, Recurrence of CDI |
| Cadena, 2010 [[36](#_ENREF_36)] | USA | Cross-sectional  retrospective | 15 month period 2003 to 2005 | Outpatients and inpatients, including long-term care, spinal injury and bone marrow transplant | Relapsing 68.76 years (SD +/- 13.86), non-relapsing 66.62 (+/- 14.23) | Positive toxin A+B assay | No | 129 | Recurrence of CDI |
| Campbell, 2009 [[23](#_ENREF_23)] | USA | Cross-sectional  retrospective | Jan 2006- Dec 2006 | Hospital inpatients and nursing homes | Not stated but included paediatric patients | Enzyme immunoassay, endoscopy, cytotoxin, histopathology | No | 14 329 | Recurrence of CDI |
| Choi, 2011 [[67](#_ENREF_67)] | S Korea | Cross-sectional  retrospective | Dec 2008 - Oct 2010 | All inpatients | Mean age 62.5 years (range 15 – 84) | Stool culture, toxin analysis or colonic or HP confirmation of pseudomembranous colitis | No | 84 | Recurrence of CDI |
| Chung, 2010 [[30](#_ENREF_30)] | Taiwan | Cross-sectional  retrospective | Jan 2007 to Mar 2008 | Adult inpatients in medical wards or in ICU | Mean age 61.1 years (range 18 – 90) | Toxin analysis by EIA | No | 86 | Recurrence of CDI |
|  |  |  |  |  |  |  |  |  |  |
| Fuji, 2010 [[46](#_ENREF_46)] | USA | Case-control  retrospective | Jan 2005 to Dec 2007 | Appendectomy and non-appendectomy patients | Mean age 67 years | ‘Positive C.*difficile* detection’ from laboratory database | No | 386 | Recurrence of CDI |
| Garey, 2010 [[29](#_ENREF_29)] | USA | Cohort  prospective | 2007-2008 | Adult inpatients | Mean age 62 years (range 49 – 75) | Cytotoxin B culture | No | 96 | Recurrence of CDI |
| Golan, 2011 [[66](#_ENREF_66)] | Not given | Clinical trial (cohort design)  prospective | 18 months (unspecified) | Patients on medicines | Adults | Toxin test | No | 518 (253 treated with fidaxomycin and 265 with vancomycin) | Recurrence of CDI |
| Grube, 1987 [[41](#_ENREF_41)] | USA | Cohort  prospective | Jul 1985-Jun 1986 | Burns patients in ICU | Mean age 32.4 (SD +/-24.6) | Culture or cytotoxin | No | 9 | Recurrence of CDI |
| Hamel, 2010 [[38](#_ENREF_38)] | Canada | Unclear design  retrospective | June 2001- Dec 2005 | Hospital inpatients | Mean age 60 years (SD +/- 20.2) | Enzyme immunoassay | No | 202 | Contact Pattern |
| Hayek, 1997 [[50](#_ENREF_50)] | Germany | Cross-sectional  retrospective | Sep 1994 - Mar 1996 | Neurology and psychiatric gerontology | Not available | ELISA assay | No | 75 | Recurrence of CDI |
| Kasper, 2010 [[65](#_ENREF_65)] | Austria | Cross-sectional  retrospective | not clear | All inpatients | Median age 80 years (range 31-98) | Not clear | Yes | 38 | Recurrence of CDI |
| Kelsen, 2011 [[28](#_ENREF_28)] | USA | Case-control  retrospective | Jan 1997-Feb 2007 | Paediatric patients with inflammatory bowel disease | Mean age cases 14.1 years (range 5 – 21 years) | Stool EIA for toxin A+B | No | 111 cases (IBD+CD), 77 controls (CD, no IBD) | Recurrence of CDI |
| Keven, 2004 [[27](#_ENREF_27)] | USA | Cross-sectional  retrospective | Jan 1999 - Dec 2002 | Adults undergoing kidney or pancreas-kidney transplantation | Mean age 53 years (range 39-67) | Positive stool toxin | No | 35 | Recurrence of CDI |
| Khan, 2012 [[26](#_ENREF_26)] | Qatar | Cross-sectional  retrospective | 2006-2009 | inpatients >=15 years | Mean age 50.9 years (range 29.7-72.1) | EIA (toxins A+B) or endoscopy  /histology showing PMC | No | 118 | Recovery Rate, Recurrence of CDI |
| Khanna, 2009 [[18](#_ENREF_18)] | USA | Cross-sectional  retrospective | 1991-2005 | Hospital inpatients and outpatients | Community acquired median age 50 years (range 1 – 102)  Hospital acquired median age 72 (range 1 month – 99 years) | Stool assay/histology/  endoscopy | No | 385 | Recurrence of CDI |
| Khanna, 2009 [[19](#_ENREF_19)] | USA | Cross-sectional  retrospective | 1991-2005 | Pediatric cases from study #64 | Median age 19 months (range 1 month – 17 years) | Stool assay/histology/  endoscopy | No | 30 children | Recurrence of CDI |
| Kim, 2010 [[24](#_ENREF_24)] | S Korea | Cross-sectional  retrospective | Jan 2006- Dec 2007 | Presumed all adult inpatients | Mean age 67.6 years (range 53.7 – 81.5) | ELISA for toxin A & B | No | 125 | Recurrence of CDI |
| Kim, 2011 [[55](#_ENREF_55)] | S Korea | Cohort  prospective | Sep 2008-Jan 2010 | Hospitalised patients | Mean age 64 years (SD +/- 15.58) | Toxin genes by PCR for diagnosis, positive toxin assay (A or B) or pseudomembrane on endoscopy  /histology | No | 140 | Recovery Rate, Recurrence of CDI |
| Kim, 2011 [[40](#_ENREF_40)] | USA | Cross-sectional  retrospective | Jan 2004-Jun 2010 | Residents in long-term care/  rehabilitation facility | Hospital acquired median age 74 years (IQR 62-82), long term care facility median age 77 years (IQR 66-83) | Stool ELISA (toxin A+B) or PCR for toxin gene | No | 162 | Recurrence of CDI |
| Kim, 2012 [[25](#_ENREF_25)] | USA | Cohort  prospective | Mar 2008-Dec 2009 | Hospitalised children | Median age 5.93 years severe CDI (1.78-12.6) and 1.83 years non severe CDI (0.67-8.1) | ELISA for toxins A+B and positive stool culture. PCR for toxin genes, +/- GDH | No | severe CDI 48  non-severe CDI 34 | Recurrence of CDI |
| Kim, 2012 [[45](#_ENREF_45)] | S Korea | Case-control  retrospective | Jan 2004-Dec 2008 | Hospitalised patients | Mean age, recurrent group: 66.8 years (SD + / - 1.0)  Mean age, non-recurrent group: 64.3 years (SD +/- 2.7) | Toxin analysis | No | 198 | Recurrence of CDI |
| Kim, 2013 [[22](#_ENREF_22)] | S Korea | Cross-sectional  retrospective | 2004-2008 | Adult hospital admissions (>18 years) | Mean age 61.6 years (SD +/- 15.3) | EIA (toxins A+B) or positive stool culture, or endoscopy | No | 1367 | Recovery Rate, Recurrence of CDI |
| Kyne, 1998 [[54](#_ENREF_54)] | Ireland | Cross-sectional  retrospective | Jan-Jun 1995 | All inpatients | Mean age 68.8 years (49.8-87.8) | Culture + cytotoxin assay (toxin B) | No | 73 | Recurrence of CDI |
| Laffan, 2006 [[64](#_ENREF_64)] | USA | Cross-sectional  retrospective | Jul 2001- Dec 2003 | Rehabilitation unit, Sub acute unit, Nursing home: | Age not stated, but data from geriatric long term care facility | Stool enzyme immunoassay for toxin A & B | No | 69 | Recurrence of CDI |
| Lanzas, 2011 [[42](#_ENREF_42)] | USA | Cross-sectional  retrospective | Jan 2008-Dec 2008 | 6 medical wards | Mean age 57 years. | Toxin analysis | No | 11046 patients, analysed, number with CDI unclear | Basic Reproduction Number |
| Lee, 2010 [[49](#_ENREF_49)] | S Korea | Cross-sectional  retrospective | Jan 2003- Dec 2008 | Hospital inpatients | Mean age 64.05 years (range 48.05-80.05) | Toxin assay,stool culture, endoscopy | No | 302 | Recurrence of CDI |
| Legaria, 2003 [[63](#_ENREF_63)] | Argentina | Cross-sectional  prospective | Apr 2000-Apr 2001 | All inpatients | Mean age 57 years (range 26-87) | EIA (toxins A+B) | No | 32 | Recurrence of CDI |
| Linsky, 2010 [[21](#_ENREF_21)] | USA | Cohort  retrospective | Oct 2003-Sep 2008 | Inpatients+  outpatients from 8 Veterans Affairs medical centers/  clinics | Median age 74 years range 63 – 82) | Stool EIA for toxins A+B | No | 1166 | Recurrence of CDI |
| Lu, 1994 [[35](#_ENREF_35)] | USA | Cohort  prospective | Apr 1992-Apr 1993 | Presumed all inpatients  +outpatients. HIV-infected and non-HIV infected patients | Mean age HIV +ve 38.5 years (SD +/-12.5), non-HIV mean age 68.8 (SD +/-19.9) | EIA for toxin A or cytotoxicity assay | No | HIV positive with CDI 32,  non-HIV with CDI 55 | Recurrence of CDI |
| Munoz, 2007 [[62](#_ENREF_62)] | Spain | Cohort study design but case-control adapted for risk factor analysis  prospective | Jan 1993- Dec 2005 | Patients who underwent heart transplant (1993-2005) | Mean age 53 years (SD +/- 9.4) | Cytotoxin assay and stool culture | No | 35 | Recurrence of CDI |
| Noren, 2004 [[34](#_ENREF_34)] | Sweden | Cross-sectional  retrospective | Feb 1999-Jan 2000 | All *C diff* cases in county (hospital and community) | Median age 74 years (range 1 – 93) | Toxin-positive C diff or toxin in stool | Yes | 330 | Contact Pattern, Basic Reproduction Number, Recurrence of CDI |
| Pawar, 2012 [[61](#_ENREF_61)] | USA | Surveillance study (cross-sectional)  prospective | Jan 2010- Dec 2010 | Long-term care facility patients | Median age 84 years (range 74-89) | Toxin enzyme immunoassay, GDH and PCR for toxin gene | No | 394 | Recurrence of CDI |
| Pepin, 2012 [[20](#_ENREF_20)] | Canada | Cross-sectional  retrospective | Jan 1998-Dec 2009 | All population of one region in Canada | Cases range 28 – 90 years, contacts 0.8 – 91 years) | Cytotoxicity assay - a few diagnosed solely by endoscopy, pathology | No | 2222 index cases | Contact Pattern, Serial Interval |
| Pizzala, 2012 [[33](#_ENREF_33)] | South America | Cohort  retrospective | Mar 2003-Dec 2010 | Inpatients and outpatients | Mean age 61.8 years – outpatients. 62.6 years inpatients | Enzyme immunoassay, cytotoxicity assay | No | 136 | Recurrence of CDI |
| Polivkova, 2010 [[48](#_ENREF_48)] | Czech Republic | Cross-sectional  retrospective | Jan 2008 -Jun 2010 | Hospital inpatients | Median age 66 years (range 5 – 88) | Enzyme linked fluorescent assay | No | 82 | Recurrence of CDI |
| Ross, 2012 [[60](#_ENREF_60)] | UK | Cross-sectional  retrospective | June 2010-May 2011 | Presumed all inpatients | Mean age 75.5 years (range 2 -100) | Positive stool toxin, also GDH in some patients | No | 56 | Recurrence of CDI |
| Samore, 1996 [[53](#_ENREF_53)] | USA | Cohort  prospective | Jun 1992-Dec 1992 | All hospital inpatients | Not stated | Stool cytotoxin assay (toxin B) | No | 52 index cases | Contact Pattern, Incubation Period |
| SanGill, 2010 [[59](#_ENREF_59)] | Costa Rica | Cross-sectional  retrospective | Jan to Oct 2009 | Multispecialty hospitals | Not stated | Immunoassay | No | 427 | Recurrence of CDI |
| Shaw,  2008 [[58](#_ENREF_58)] | USA | Cohort  retrospective | Jan 2006- Dec 2006 | Hospital inpatients | Not stated | Positive immunosorbent assay | No | 201 | Recurrence of CDI |
| Van Der Zwet , 2012 [[39](#_ENREF_39)] | Nether-lands | Cohort  prospective | Dec 2010 - Feb 2011 | Surgery/  gastroenterology patients | Mean age 77 years (range 64-88) | Toxin assay, immunoanalysis of faecal samples, PCR for diagnosis ribotyping | Yes (ribotyping) | 20 | Recurrence of CDI |
| Vesteinsdottir, 2012 [[32](#_ENREF_32)] | Iceland | Case-control  prospective | Jul 2010-Jun 2011 | All population of Iceland | Median age 68 years (IQR 56-80) | ELISA for toxins A+B | No | 103 | Recovery Rate, Recurrence of CDI |
| Vojtilovia, 2011 [[47](#_ENREF_47)] | Not clear | Cross-sectional  retrospective | Jan2007 - Dec 2010 | Hospital inpatients | Mean age 73.2 years | Cytotoxin, endoscopy | Yes (limited to 4 patients) | 284 | Recurrence of CDI |
| Waggoner, 1994 [[44](#_ENREF_44)] | USA | Case-control  retrospective | Aug 1986-Jan 1989 | Gynaecologic oncology | Mean age cases 61 years (range 30-76), controls 59 years (range 29-87) | Latex agglutination CDT (antigen detection) | No | 23 | Recurrence of CDI |
| Walker, 2012 [[2](#_ENREF_2)] | UK | Cross-sectional  retrospective | Sep 2007-Mar 2010 | All inpatients, outpatients, primary care | Not stated | EIA (toxins A+B) | Yes (MLST) | 218 | Incubation Period, Serial Interval |
| Yagues, 2001 [[57](#_ENREF_57)] | Not given | Cross-sectional  retrospective | Nov 1997-Oct 1999 | Transplant and non-transplant patients | Not stated | Toxin positive stool | No | 375 | Recurrence of CDI |
