# Supplementary material for: Parameters for the Mathematical Modelling of Clostridium difficile Acquisition and Transmission: A Systematic Review
Source: PLoS One. 2013 Dec 20;8(12):e84224. doi: 10.1371/journal.pone.0084224 (PMC3869946; doi:10.1371/journal.pone.0084224)
Supplement: Table S3 — Studies reporting data on recurrence rate. (DOCX) [file pone.0084224.s003.docx]

**Table S3: Studies reporting data on recurrence rate (n=49)**

| **Author** | **Year** | **Number analysed** | **Recurrence Rate – (% rounded to nearest integer)** | **Definition used for recurrence** | **Group studied** |
| --- | --- | --- | --- | --- | --- |
| Alanganden [[70](#_ENREF_70)] | 2000 | 13 | 15% | relapse rate (not specified but presumed lab diagnosed) in allogeneic stem cell transplantation recipients | Hemopoietic stem cell transplantation |
| Alanzai [[31](#_ENREF_31)] | 2012 | 83 | 8% | Recurrent CDI: unclear whether lab diagnosed | Pediatric patients undergoing hemopoietic stem cell transplantation |
| Armbruster [[37](#_ENREF_37)] | 2012 | 188 | 14% | Recurrence rate in patients treated with metronidazole | Hospital inpatients and outpatients |
|  |  | 22 | 9% | Recurrence rate in patients treated with vancomycin |  |
| Aronsson [[52](#_ENREF_52)] | 1987 | 62 | 26% | Relapse of diarrhoea | Patients with acute or chronic renal failure |
| Barany [[69](#_ENREF_69)] | 1992 | 70 | 31% | Episodes of relapse | Chronic renal failure (n=62) acute renal failure(n=8) |
| Beausejour [[43](#_ENREF_43)] | 2010 | 86 | 36% | CDAD relapse | Presumed all hospital inpatients but not clear |
| Berling [[68](#_ENREF_68)] | 2009 | 40 | 20% | Relapse or re-infection, however data not clearly stated. | Nursing homes |
| Bilgrami [[56](#_ENREF_56)] | 1999 | 14 | 7% | Relapse rate | Blood stem cell transplant patients |
| Bouza [[51](#_ENREF_51)] | 1995 | 120 | 6 % | Relapse rate | Hospital inpatients |
| Cadena [[36](#_ENREF_36)] | 2010 | 129 | 29% | Relapsing CDI: recurrent symptoms of CDI (diarrhea and positive stool toxin assay) after completion of therapy and initial resolution | Outpatients and inpatients, including long-term care, spinal injury and bone marrow transplant |
| Campbell [[23](#_ENREF_23)] | 2009 | 14329 |  | Healthcare onset recurrent case = any initial case patient who had positive test results, endoscopy or biopsy within 6 months of initial episode. | Hospital inpatients and nursing homes |
|  |  |  | 1.1-2.0 cases / 1000 patient days | Rate of recurrence in hospitals |  |
|  |  |  | 0.8-2.4 cases / 1000 patient days | Rate of recurrence in nursing homes |  |
| Choi [[67](#_ENREF_67)] | 2011 | 84 | 13% | Recurrence of symptoms or positive assay within 60 days of cure | Hospital inpatients |
| Chung [[30](#_ENREF_30)] | 2010 | 86 | 8% | Recurrence rate | Adult inpatients in medical wards or in ICU |
| Fujii [[46](#_ENREF_46)] | 2010 | 61 | 26% | Relapse rate – patients with prior appendectomy | Patients with or without prior appendectomy |
|  |  | 325 | 22% | Relapse rate – patients without prior appendectomy |  |
| Garey [[29](#_ENREF_29)] | 2010 | 96 | 24% | Recurrence: a new episode of diarrhea lasting at least 2 days, with a positive toxin assay test result, after resolution of the initial episode and after discontinuation of antibiotics | Adult hospital inpatients |
| Golan [[66](#_ENREF_66)] | 2011 | 518 |  | Recurrence: appearance of diarrhoea and a positive stool toxin test within 4 weeks following completion of therapy. | Adults receiving fidaxomicin or vancomycin as part of a clinical trial |
|  |  |  | 21% (10.5 recurrences / 1000 patient days) | Recurrence Rate (all patients) |  |
|  |  |  | 5.9 recurrences/1000 patient days | Recurrence Rate (with fidaxomicin) |  |
|  |  |  | 15.1 recurrences/1000 patient days | Recurrence Rate (with vancomycin) |  |
| Grube [[41](#_ENREF_41)] | 1987 | 9 | 22% | Recurrence rate (lab diagnosed) | Burns patients in ICU |
| Hayek [[50](#_ENREF_50)] | 1997 | 75 | 35% | Relapse rate not otherwise specified, unclear if lab diagnosed | Neurology and psychiatric gerontology |
| Kasper [[65](#_ENREF_65)] | 2010 | 38 | 16% | Recurrence: second episode of CDI within 60 days of the first episode | Hospital inpatients with CDI 027 |
| Kelsen [[28](#_ENREF_28)] | 2011 | 111 IBD patients | 34% | Recurrence: separate episode of diarrhoea with toxin-positive stool within 60 days of prior infection, with at least one stool showing clearance between infections. Minimum repeat time for testing 2 weeks | Pediatric patients with / without inflammatory bowel disease |
|  |  | 77 non-IBD patients | 8% |  |  |
| Keven [[27](#_ENREF_27)] | 2004 | 35 | 23% | Recurrent infection but not clear whether lab diagnosed | Adults undergoing kidney or pancreas-kidney transplantation |
| Khan [[26](#_ENREF_26)] | 2012 | 118 | 10% | Recurrence of toxin positive diarrhea within 60 days from initial diagnosis | Inpatients ≥ 15 years |
| Khanna [[18](#_ENREF_18)] | 2009 |  |  | Recurrence: recurrent infection within 8 weeks of successful treatment | Hospital inpatients and outpatients from one county |
|  |  | 158 | 29% | Recurrence rate in community-acquired cases |  |
|  |  | 192 | 30% | Recurrence rate in hospital-acquired cases |  |
| Khanna [[18](#_ENREF_18),[19](#_ENREF_19)] | 2009 | 22 | 22% | Recurrence rate after metronidazole treatment | Hospital inpatients and outpatients from one county |
|  |  | 5 | 20% | Recurrence rate after vancomycin treatment |  |
| Kim [[24](#_ENREF_24)] | 2010 | 125 | 22% | Recurrence: diarrhoea recurrence with a positive ELISA test within 90 days of therapy completion, and after complete resolution of signs and symptoms (all patients) | Presumed adult hospital inpatients |
|  |  |  | 21% | Recurrence rate within 90 days of cure in patients treated with metronidazole |  |
|  |  |  | 17% | Recurrence rate within 90 days of cure in patients treated with vancomycin |  |
| Kim [[55](#_ENREF_55)] | 2011 | 140 | 21 % | Recurrence (lab diagnosed) ≥10 days after 1st episode | Hospital inpatients |
| Kim [[40](#_ENREF_40)] | 2011 |  |  | Relapse: recurrence of diarrhoea after initial cure with or without positive *C. difficile* toxin, within 6 months. | Residents in long-term care / rehabilitation facility |
|  |  | 66 | 49% | Hospital associated CDI |  |
|  |  | 96 | 29% | Long term care facility associated CDI |  |
| Kim [[25](#_ENREF_25)] | 2012 | 82 | 24% | Relapse: any recurrence of CDI symptoms within 8 weeks of follow-up or receipt of an additional course of antibiotics > 2 weeks after completion of initial course (all children) | Hospitalised children |
|  |  | 48 | 31% | Relapse (as defined above) in children with severe CDI |  |
| Kim [[45](#_ENREF_45)] | 2012 | 198 | 14% | Recurrence rate | Hospitalised patients |
| Kim [[22](#_ENREF_22)] | 2012 | 1367 | 9% | Relapse rate (≥ 8 weeks after symptom resolution). Not lab confirmed | Adult hospital admissions (>18 years) |
| Kyne [[54](#_ENREF_54)] | 1998 | 73 | 27% | ‘Relapsing disease’ | Hospital inpatients |
| Laffan [[64](#_ENREF_64)] | 2006 | 69 | 22% | Recurrence: *C.difficile* positive EIA test > 7days after previous positive result | Rehabilitation unit, sub-acute unit and nursing home unit of long-term care facility |
| Lee [[49](#_ENREF_49)] | 2010 | 69, | 13%, | Recurrence rates 2003-2005. Recurrence rates 2006-2008 | Hospital inpatients |
|  |  | 233 | 12% |  |  |
| Legaria [[63](#_ENREF_63)] | 2003 | 32 | 16% | Symptomatic recurrence (resurgence of symptoms more than 10 days later) | Hospital inpatients |
| Linsky [[21](#_ENREF_21)] | 2010 | 1166 | 22% | Recurrence rate between 12 and 90 days | Inpatients and outpatients from 8 Veterans Affairs Medical Centers / clinics |
| Lu [[35](#_ENREF_35)] | 1994 |  |  | Recurrence: renewed diarrhoea, fever, leukocytosis or abdominal pain with positive stool assay after discontinuing therapy | Presumed all inpatients and outpatients. HIV-infected compared with non-HIV infected patients |
|  |  | 32 | 9% | Recurrence rate in HIV positive patients after 5-10 days of therapy. |  |
|  |  | 55 | 15% | Recurrence rate in non-HIV patients after 5-10 days of therapy. |  |
| Munoz [[62](#_ENREF_62)] | 2007 | 35 | 29% | Recurrence rate | Patients who underwent heart transplant between (1993-2005) |
| Noren [[34](#_ENREF_34)] | 2004 | 267 | 25% | Recurrence: New episode with onset 2 weeks or more after primary episode. Relapse: recurrence due to the same genotype as in the initial episode. Re-infection: recurrence due to new type of strain. Isolates showed 90% of ‘recurrences’ were relapse, 10% were re-infection | All CDI cases in county (hospital and community) |
| Pawar [[61](#_ENREF_61)] | 2012 | 394 | 1.0 cases per 10,000 resident-days (IQR: 0.3-1.4) | Recurrence: a stool specimen positive for *C.difficile* obtained between 2-8 weeks after the last *C.difficile*-positive stool specimen. Based on the total number of recurrent incidents any one patient may have contributed | Long-term care facility patients |
| Pizzala [[33](#_ENREF_33)] | 2012 | 136 | 19% | Recurrences in outpatients | Hospital inpatients and outpatients |
|  |  |  | 21% | Recurrences in inpatients |  |
| Polivkova [[48](#_ENREF_48)] | 2010 | 82 | 21% | Stated as recurrence (fully translated paper unavailable) | Hospital inpatients |
| Ross [[60](#_ENREF_60)] | 2012 | 56 | 7% | Recurrence rate | Presumed hospital inpatients |
| San Gill [[59](#_ENREF_59)] | 2010 | 427 | 3% | 11/14 recurrences were due to relapse, 3 to reinfection | Multispecialty hospitals |
| Shaw [[58](#_ENREF_58)] | 2008 | 201 | 20% | Recurrence rate | Hospital inpatients |
| Van Der Zwet [[39](#_ENREF_39)] | 2012 | 20 | 45% | Relapse rate | Surgery / gastroenterology patients |
| Vesteinsdottir [[32](#_ENREF_32)] | 2012 | 103 | 22% | Relapse: a further positive stool sample within 3 months | All population of Iceland |
| Vojtilova [[47](#_ENREF_47)] | 2011 | 284 | 31% | Recurrence rate | Hospital inpatients |
| Waggoner [[44](#_ENREF_44)] | 1994 | 23 | 26% | Relapse rate | Gynecologic oncology patients |
| Yagues [[57](#_ENREF_57)] | 2001 | 375 | 23% | Relapse rate in transplant patients. | Transplant and non-transplant patients |
|  |  |  | 15% | Relapse rate in non-transplant patients |  |

**Table S4: Parameter definitions [**[**16**](#_ENREF_16)**]**

| **Parameter term** | **Definition** |
| --- | --- |
| Basic reproduction number (*R_0_*) | The basic reproduction number is the average number of secondarily infected individuals that result from one infected individual in a totally non immune (susceptible) population. |
| Contact pattern | Describes the interaction of individuals which may lead to infection. These may be random or non-random and include the rate of contact, the average number of contacts and the amount of mixing. |
|  |  |
| Duration of infectiousness (*D*) | Describes the length of time that infected individuals remain capable of transmitting the infection |
| Force of infection (incidence or hazard rate) (λ_t_) | Describes the rate at which susceptible individuals are infected by time |
| Incubation period | Describes the duration (time) between being infected and the onset of symptoms, although for some diseases, individuals can be infectious while remaining asymptomatic or will be infectious before symptoms present |
| Net reproduction number (effective reproduction number) (*R* or *R_n_*) | The basic reproduction number is the average number of secondarily infected individuals that result from one infected individual in a partially immune population (from prior infection or immunization). |
| Recovery rate (*r*) | Describes the proportion of individuals that recover from their infection over time |
| Recurrence rate | Describes the rate at which individuals move from having recovered from their infection to being susceptible again |
| Serial interval (generation time) | Describes the time to produce the next infection in a transmission chain |
