# Supplementary material for: Parameters for the Mathematical Modelling of Clostridium difficile Acquisition and Transmission: A Systematic Review
Source: PLoS One. 2013 Dec 20;8(12):e84224. doi: 10.1371/journal.pone.0084224 (PMC3869946; doi:10.1371/journal.pone.0084224)
Supplement: Table S4 — Parameter definitions. (DOCX) [file pone.0084224.s004.docx]

**Table S4: Parameter definitions [**[**16**](#_ENREF_16)**]**

| **Parameter term** | **Definition** |
| --- | --- |
| Basic reproduction number (*R_0_*) | The basic reproduction number is the average number of secondarily infected individuals that result from one infected individual in a totally non immune (susceptible) population. |
| Contact pattern | Describes the interaction of individuals which may lead to infection. These may be random or non-random and include the rate of contact, the average number of contacts and the amount of mixing. |
|  |  |
| Duration of infectiousness (*D*) | Describes the length of time that infected individuals remain capable of transmitting the infection |
| Force of infection (incidence or hazard rate) (λ_t_) | Describes the rate at which susceptible individuals are infected by time |
| Incubation period | Describes the duration (time) between being infected and the onset of symptoms, although for some diseases, individuals can be infectious while remaining asymptomatic or will be infectious before symptoms present |
| Net reproduction number (effective reproduction number) (*R* or *R_n_*) | The basic reproduction number is the average number of secondarily infected individuals that result from one infected individual in a partially immune population (from prior infection or immunization). |
| Recovery rate (*r*) | Describes the proportion of individuals that recover from their infection over time |
| Recurrence rate | Describes the rate at which individuals move from having recovered from their infection to being susceptible again |
| Serial interval (generation time) | Describes the time to produce the next infection in a transmission chain |
